# Supplementary material for: Molecular sorting of nitrogenase catalytic cofactors[image]
Source: J Biol Chem. 2025 Feb 10;301(3):108291. doi: 10.1016/j.jbc.2025.108291 (PMC11938142; doi:10.1016/j.jbc.2025.108291)
Supplement: Table S1 [file mmc2.docx]

**Table S1. List of *Azotobacter vinelandii* strains.**

| **Strain** | **Genotype** |
| --- | --- |
| DJ2239 | *ΔnifDK*, *vnfDGK*::*sm^R^*,*rif*^R^, Δ42kb^a^ |
| DJ2240 | *ΔnifDK*, *vnfDGK*::*sm^R^*, *anfDGK*::*km^R^, rif^R^*, Δ42kb^a^ |
| DJ2241 | *ΔnifDK*, *vnfDGK*::*sm^R^*, *anfD^S-TAG^, rif^R^*, Δ42kb^a^ |
| DJ2245 | *ΔnifDK*, *vnfDGK*::*sm^R^*, Δ*nifB*::*km^R^, anfD^S-TAG^, rif^R^*, Δ42kb^a^ |
| DJ2290 | *ΔnifDK*, *vnfDGK*::*sm^R^*, Δ*anfO*, *anfD^S-TAG^, rif^R^*, *tet^R^*, Δ42kb^a^ |
| DJ2494 | *ΔnifDK*, *vnfDGK*::*sm^R^*, *anfO^S-TAG^* ^(C-term)^, *rif^R^*, *tet^R^*, Δ42kb^a^ |
| DJ2520 | *ΔnifDK*, *vnfDGK*::*sm^R^*, Δ*nifB,* *rif*^R^, Δ42kb^a^ |
| DJ2527 | *ΔnifDK*, *vnfDGK*::*sm^R^*, *anfO^S-TAG^* ^(N-term)^, *rif^R^*, *tet^R^*, Δ42kb^a^ |
| DJ2560 | *ΔnifDK*, *vnfDGK*::*sm^R^*, *vnfE*::*gm^R^*, Δ*modE1*^a^, *anfD^S-TAG^, rif^R^* |
| DJ2821 | *ΔnifDK*, *vnfDGK*::*sm^R^*, *vnfE*::*gm^R^*, Δ*modE1*^a^, Δ*anfO*, *anfD^S-TAG^, rif^R^* |
| DJ2831 | *ΔnifDK*, *vnfDGK*::*sm^R^*, *vnfE*::*gm^R^*, Δ*modE1*^a^, Δ*anfO*, *nifE*::*km^R^, anfD^S-TAG^, rif^R^* |
| DJ2911 | *ΔnifDK*, *vnfDGK*::*sm^R^, anfD^S-TAG^, anfO^C159A^*, Δ42kb^a^ |
| DJ2912 | *ΔnifDK*, *vnfDGK*::*sm^R^, anfD^S-TAG^, anfO^H203L^*, Δ42kb^a^ |
| DJ2916 | *ΔnifDK*, *vnfDGK*::*sm^R^, anfD^S-TAG^, anfO^C201A^*, Δ42kb^a^ |

*anfD^S-TAG^*: Strep-tag is placed at the C-terminal of *anfD; rif^R^*: rifampicin; *sm^R^*: streptomycin; *km^R^*: kanamycin; *tet^R^*: tetracycline; *gm^R^*: gentamycin.

Location of residues removed and/or placement of insertions are indicated in Table S2.

^a^ W-tolerance is the result of a Δ42kbp in a genomic deletion required for Mo acquisition or the Δ*modE1* whose product is involved in regulating Mo acquisition and Mo-dependent repression of *anf* gene expression.
