# Supplementary material for: Molecular sorting of nitrogenase catalytic cofactors[image]
Source: J Biol Chem. 2025 Feb 10;301(3):108291. doi: 10.1016/j.jbc.2025.108291 (PMC11938142; doi:10.1016/j.jbc.2025.108291)
Supplement: Table S2 [file mmc3.docx]

**Table S2. List of plasmids used for the construction of *Azotobacter vinelandii* strains**.

Location of residues removed and/or placement of insertions are indicated. Nomenclature corresponds to the genotype shown in Supplemental Table 1. S-TAG: Strep-tag (ASWSHPQFEK); km^R^: kanamycin; sm^R^: streptomycin; gm^R^: gentamycin.

**Plasmid Deletion/Insertion Residues Removed/**

**Insertion Location**

pDB33 Δ*nifDK* NifD^103^ - NifK^308^

pDB161 Δ*nifB* NifB^60-307^

pDB218 *nifB*::km^R^ NifB^60-307^

pDB259 *nifE*::km^R^ NifE^15-261^

pDB2134 *anfDGK*::km^R^: AnfD^204^ - AnfK^148^

pDB2139 *vnfDGK*::sm^R^ VnfD^271^ - VnfK^202^

pDB2158 *anfD^S-TAG^* AnfD^518^

pDB2200 *vnfE*::gm^R^ VnfE^91^

pDB2224 Δ*anfO* AnfO^179-224^

pDB2265 Δ*modE1* ModE1^151-215^

pDB2355 *anfO^S-TAG^* ^(C-term)^ AnfO^245^

pDB2395 *anfO^S-TAG^* ^(N-term)^ AnfO^1^

pDB2611 AnfO^C159A^

pDB2612 AnfO^C201A^

pDB2613 AnfO^H203L^
