# Supplementary material for: Molecular sorting of nitrogenase catalytic cofactors[image]
Source: J Biol Chem. 2025 Feb 10;301(3):108291. doi: 10.1016/j.jbc.2025.108291 (PMC11938142; doi:10.1016/j.jbc.2025.108291)
Supplement: Table S3 [file mmc4.docx]

**Table S3. Plasmids used for heterologous expression of AnfO and the N- and C-domains in *Escherichia coli* BL21(DE3) competent cells.**

| **Plasmid** | **Description** |
| --- | --- |
| pDB2343 | For purification of the non-tagged, full-length AnfO (residues 1-245). |
| pDB2418 | For purification of the full-length AnfO (residues 1-245). A Strep-tag (ASWSHPQFEK) is located after residue 245. |
| pDB2526 | For purification of the C-terminal domain of AnfO (residues 138-245). A TwinStrep-tag (ASWSHPQFEKGGGSGGGSGGSAWSHPQFEKAS) is located before residue 138. |
| pDB2554 | For purification of the N-terminal domain of AnfO (residues 1-132). A Strep-tag (ASWSHPQFEK) is located after residue 132. |
|  |  |
