# Supplementary material for: Molecular sorting of nitrogenase catalytic cofactors[image]
Source: J Biol Chem. 2025 Feb 10;301(3):108291. doi: 10.1016/j.jbc.2025.108291 (PMC11938142; doi:10.1016/j.jbc.2025.108291)
Supplement: Table S4 [file mmc5.docx]

**Table S4. Quantification of FeMo-cofactor binding to AnfO based ICP-MS metal and BCA protein assays.**

| **Sample** | **Fe** | **Mo** | **AnfO** | **[Mo] : [AnfO]** |
| --- | --- | --- | --- | --- |
| 1 | 8.4 | 1 | 1.6 | 0.62 |
| 2 | 7.1 | 1 | 2.3 | 0.44 |
| 3 | 6.6 | 1 | 1.9 | 0.53 |
| 4 | 11 | 1 | 2.2 | 0.45 |
| **Combined** | **7.1** | **1** | **2.0** | **0.51** |

The “Combined” sample was prepared by mixing samples 1-4 and concentration using a stirred cell concentrator. These data show that repeated generation of AnfO containing FeMo-cofactor results in an [Fe] : [Mo] ratio close to 7:1, as is expected for a FeMo-cofactor containing protein. Some occurrences of higher [Fe] : [Mo] ratio have been observed (notably sample 4), which we attribute to cluster degradation. Additionally, the [Mo] : [AnfO] ratio, which we use as a proxy for the [FeMo-cofactor] : [AnfO] ratio, consistently resides in the range of 0.5 ± 0.1. At this time, we cannot distinguish between weak FeMo-cofactor binding to AnfO (and a native 1:1 cofactor:AnfO ratio) or a native 1:2 cofactor:AnfO ratio.
